# Imaging Modality and Frequency in Surveillance of Stage I Seminoma Testicular Cancer: Results From a Randomized, Phase III, Noninferiority Trial (TRISST)

**DOI:** 10.1200/JCO.21.01199

**Published:** 2022-03-17

**Authors:** Johnathan K. Joffe, Fay H. Cafferty, Laura Murphy, Gordon J.S. Rustin, Syed A. Sohaib, Rhian Gabe, Sally P. Stenning, Elizabeth James, Dipa Noor, Simona Wade, Francesca Schiavone, Sarah Swift, Elaine Dunwoodie, Marcia Hall, Anand Sharma, Jeremy Braybrooke, Jonathan Shamash, John Logue, Henry H. Taylor, Ivo Hennig, Jeff White, Sarah Rudman, Jane Worlding, David Bloomfield, Guy Faust, Hilary Glen, Rachel Jones, Michael Seckl, Graham MacDonald, Thiagarajan Sreenivasan, Satish Kumar, Andrew Protheroe, Ramachandran Venkitaraman, Danish Mazhar, Victoria Coyle, Martin Highley, Tom Geldart, Robert Laing, Richard S. Kaplan, Robert A. Huddart

**Affiliations:** 1 St James University Hospital, Leeds, United Kingdom; 2 MRC Clinical Trials Unit at UCL, London, United Kingdom; 3 Mount Vernon Hospital, Northwood, United Kingdom; 4 Institute of Cancer Research, Royal Marsden Hospital, Sutton, United Kingdom; 5 Centre for Cancer Prevention, Queen Mary University of London, London, United Kingdom; 6 Hillingdon Hospital, Uxbridge, United Kingdom; 7 Bristol Haematology & Oncology Centre, University Hospitals Bristol and Weston NHS Foundation Trust, Bristol, United Kingdom; 8 Barts Cancer Institute, St Bartholomews Hospital, London, United Kingdom; 9 The Christie Hospital, Manchester, United Kingdom; 10 Kent Oncology Centre, Maidstone Hospital, Maidstone, United Kingdom; 11 Nottingham University Hospitals NHS Trust, Nottingham, United Kingdom; 12 Beatson West of Scotland Cancer Centre, Glasgow, United Kingdom; 13 Guy’s & St Thomas’ NHS Foundation Trust, London, United Kingdom; 14 University Hospital Coventry and Warwickshire, Coventry, United Kingdom; 15 Royal Sussex County Hospital, Sussex Cancer Centre, Brighton, United Kingdom; 16 Northampton General Hospital, Northampton, United Kingdom; 17 University Hospital Ayr, Ayr, United Kingdom; 18 Singleton Hospital, Swansea, United Kingdom; 19 Charing Cross Hospital, London, United Kingdom; 20 Aberdeen Royal Infirmary, Aberdeen, United Kingdom; 21 Lincoln County Hospital, Lincoln, United Kingdom; 22 Pilgrim Hospital, Boston, United Kingdom; 23 Velindre Hospital, Cardiff, United Kingdom; 24 Churchill Hospital, Oxford, United Kingdom; 25 Ipswich Hospital, Ipswich, United Kingdom; 26 Addenbrooke’s Hospital, Cambridge, United Kingdom; 27 Belfast City Hospital, Belfast, United Kingdom; 28 Derriford Hospital, Plymouth, United Kingdom; 29 University Hospitals Dorset, Poole, United Kingdom; 30 Royal Surrey County Hospital, Guildford, United Kingdom

## Abstract

**Purpose:**

Survival in stage I seminoma is almost 100%. Computed tomography (CT) surveillance is an international standard of care, avoiding adjuvant therapy. In this young population, minimizing irradiation is vital. The Trial of Imaging and Surveillance in Seminoma Testis (TRISST) assessed whether magnetic resonance images (MRIs) or a reduced scan schedule could be used without an unacceptable increase in advanced relapses.

**Methods:**

A phase III, noninferiority, factorial trial. Eligible participants had undergone orchiectomy for stage I seminoma with no adjuvant therapy planned. Random assignment was to seven CTs (6, 12, 18, 24, 36, 48, and 60 months); seven MRIs (same schedule); three CTs (6, 18, and 36 months); or three MRIs. The primary outcome was 6-year incidence of Royal Marsden Hospital stage ≥ IIC relapse (> 5 cm), aiming to exclude increases ≥ 5.7% (from 5.7% to 11.4%) with MRI (*v* CT) or three scans (*v* 7); target N = 660, all contributing to both comparisons. Secondary outcomes include relapse ≥ 3 cm, disease-free survival, and overall survival. Intention-to-treat and per-protocol analyses were performed.

**Results:**

Six hundred sixty-nine patients enrolled (35 UK centers, 2008-2014); mean tumor size was 2.9 cm, and 358 (54%) were low risk (< 4 cm, no rete testis invasion). With a median follow-up of 72 months, 82 (12%) relapsed. Stage ≥ IIC relapse was rare (10 events). Although statistically noninferior, more events occurred with three scans (nine, 2.8%) versus seven scans (one, 0.3%): 2.5% absolute increase, 90% CI (1.0 to 4.1). Only 4/9 could have potentially been detected earlier with seven scans. Noninferiority of MRI versus CT was also shown; fewer events occurred with MRI (two [0.6%] *v* eight [2.6%]), 1.9% decrease (–3.5 to –0.3). Per-protocol analyses confirmed noninferiority. Five-year survival was 99%, with no tumor-related deaths.

**Conclusion:**

Surveillance is a safe management approach—advanced relapse is rare, salvage treatment successful, and outcomes excellent, regardless of imaging frequency or modality. MRI can be recommended to reduce irradiation; and no adverse impact on long-term outcomes was seen with a reduced schedule.

## Introduction

Half of testicular tumors are seminoma.^1^ For early-stage disease, survival following orchiectomy is approximately 100%, regardless of management.^2^ Although adjuvant radiotherapy effectively reduces relapses, use has declined dramatically in recent decades because of concerns over long-term toxicity and emergence of alternative approaches.^3–6^ Use of adjuvant carboplatin, shown to be as effective for reducing relapses, has increased.^7,8^ However, given 80%-85% will not relapse, and relapses are generally treated successfully, risk of overtreatment is clear.^9^ Therefore, surveillance, on the basis of periodic cross-sectional imaging, tumor markers, and clinical examination, is now recommended in international guidelines, often as the preferred approach, particularly for lower-risk patients.^10–13^

Despite increasing adoption of surveillance, there is no evidence base to inform optimal modality and frequency of imaging. Schedules vary widely, and guidelines are not specific. Lower expression of serum tumor markers and greater variability in timing of relapse (compared with nonseminoma) raise concerns about reducing intensity and/or duration of radiologic surveillance. However, risk of second malignancy from a single chest, abdominal, and pelvic computed tomography (CT) is approximately 1/2000.^14,15^ In a 2009 survey of UK management practices, the most common surveillance schedule used seven CT scans over 5 years,^3^ a risk of 1/300 of second malignancy related to imaging. In these young patients, unlikely to die from seminoma, avoiding unnecessary radiation exposure is vital.

TRISST (ISRCTN65987321) sought to evaluate whether scan frequency could be reduced, or CT replaced with magnetic resonance imaging (MRI), without an unacceptable increase in advanced relapses.

## Methods

### Study Design

**Fig 1:**
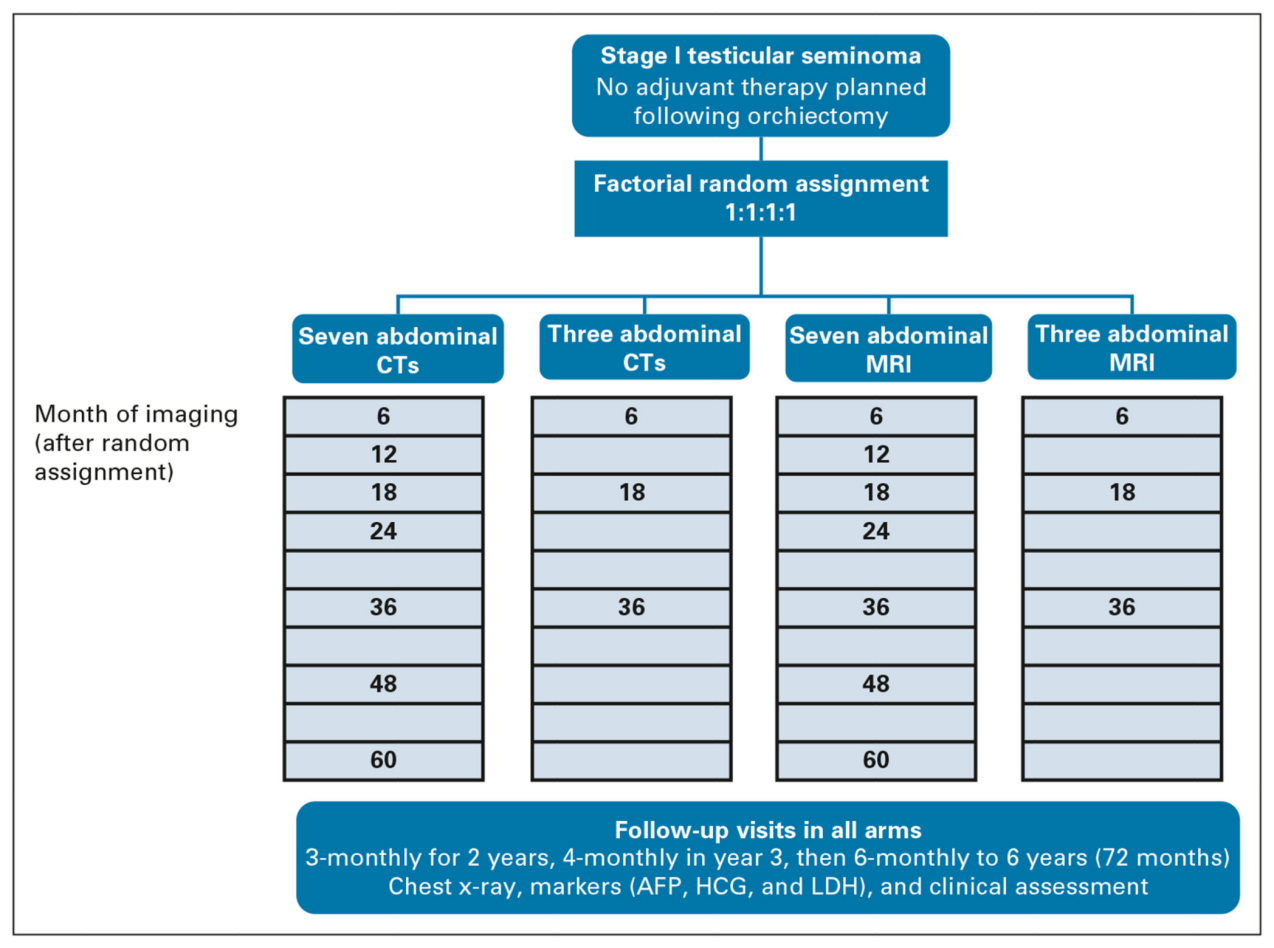
TRISST trial schema. AFP, alpha-fetoprotein; CT, computed tomography; HCG, human chorionic gonadotropin; LDH, lactate dehydrogenase; MRI, magnetic resonance imaging.

TRISST is a phase III, multicenter, open-label, randomized, noninferiority trial with 2 × 2 factorial design. Allocation, using minimization with a random element (1:1:1:1), was to seven CTs, three CTs, seven MRIs, or three MRIs of the retroperitoneum (Fig 1). Seven-scan schedules involved imaging 6, 12, 18, 24, 36, 48, and 60 months after random assignment, a common schedule in the United Kingdom when the trial was developed.^6^ Three-scan schedules involved imaging 6, 18, and 36 months after random assignment. Minimization factors were center, maximum tumor diameter (≤ 4 cm, > 4 cm), and presence of rete testis invasion. Central allocation by the trials unit ensured allocation concealment.

### Patients

Eligible patients, recruited at UK hospitals/cancer centers, were age ≥ 16 years; had histologically confirmed stage I testicular seminoma; orchiectomy < 10 weeks before random assignment; normal postorchiectomy serum alpha-fetoprotein (AFP) and Β-human chorionic gonadotropin (AFP not known to be raised preorchiectomy); and no adjuvant therapy planned. Patients with bilateral seminoma were eligible. Exclusions were coexistent of previously treated malignancy; inability to comply with assessments; contraindication to MRI; and spermatocytic tumors. Regulatory, national, and local ethical approvals were obtained. Participants provided written informed consent.

### Imaging and Follow-Up

Imaging of the retroperitoneum was performed according to allocation. Where there was a history of ipsilateral inguinoscrotal surgery, the pelvis was also imaged. Minimum imaging requirements were: CT using spiral or multi-detector scanner with a maximum reconstructed slice thickness of 5 mm and images acquired after oral and intravenous contrast media injection in the portal venous phase; MRI on at least a 1 Tesla system with phased array coils and contiguous axial 5-mm section T1 and T2 weighted images. Additional images/sequences were acquired at local radiologists’ discretion. Node measurement was taken on axial section only.

In all arms, follow-up visits took place 3-monthly in years 1-2, 4-monthly in year 3, and 6-monthly thereafter, to 6 years. Visits included clinical examination, chest x-ray (CXR), tumor markers, and imaging according to allocation.

### Relapse Detection and Treatment

Relapsing patients underwent chest CT. Those with relapse detected by markers, CXR, clinical examination, or symptoms underwent retroperitoneal CT or MRI (according to allocation). Those with relapse detected on MRI underwent confirmatory CT (within 2 weeks), providing comparative tumor measurements. IGCCCG (International Germ Cell Consensus Classification Group) intermediate-risk patients^16^ underwent brain CT and/or bone scan where indicated. Relapses were staged according to Royal Marsden Hospital (RMH) criteria.^17^ Scans between baseline and relapse underwent independent central review (to be reported separately).

Relapse treatment was at the discretion of the investigator but, for limited-stage disease (< 5 cm), recommended approach was carboplatin area under the curve 7 followed by para-aortic radiotherapy.^18^ For more advanced disease, three or four bleomycin, etoposide, and cisplatin (BEP) cycles (or four cycles of etoposide and cisplatin) were recommended, according to IGCCCG group. Follow-up continued for a minimum of 6 years after random assignment.

### Outcomes

The primary outcome was relapse with RMH stage ≥ IIC disease (para-aortic nodes > 5 cm or more extensive metastatic disease equivalent to TNM T_any_ N3 M0 or T_any_ N_any_ M1 disease). For clarity, ≥ IIC disease is used in the text, chosen to reflect a common threshold for giving BEP when the trial was designed. A 2020 survey of UK practice confirmed the relevance of this threshold, but also indicated an alternative: tumor size ≥ 3 cm.^19^ This was, therefore, prespecified as a key secondary outcome before analysis. For both, 6-year incidence was evaluated, with censoring for patients who did not experience the event, or died from another cause during follow-up. The 6-year time point ensured inclusion of any relapses missed because of the omission of the 60-month scan (in 3-scan arms), which might arise clinically thereafter.

Other secondary outcomes were mean abdominal mass size at relapse (on CT); method of detecting relapse; IGCCCG prognostic group at relapse; new primary malignancy; disease-free survival (DFS); and overall survival (OS).

### Statistical Analysis

There were two comparisons (CT *v* MRI; three *v* seven scans), with all patients contributing to both. Assuming that the population would largely comprise patients with one or no risk factors,^20^ it was expected that 15% would relapse and 38% of relapses would be RMH stage ≥ IIC.^8,21^ This equates to 5.7% of the randomized cohort. The study was designed to exclude an increase of ≥ 5.7% (noninferiority margin), to ≥ 11.4%, through a move to MRI or less frequent scanning. Treating the primary outcome as a binary measure, an estimated 660 patients were needed to achieve 80% power on the basis of 90% CIs (ie, one-sided 5% significance level, reflecting the noninferiority design) and allowing for dropout. During the follow-up period of the trial, it was decided (and prespecified with approval from oversight committees) that time-to-event analysis would facilitate incorporation of partial data from patients who did not complete follow-up. Additionally, a revision to the sample size software used suggested the original sample size was overestimated. Both of these factors meant the trial was likely to have more than 80% power.

The primary and key secondary outcomes are assessed using both intent-to-treat (ITT) and per-protocol (PP) analyses (Appendix Table A1, online only); noninferiority was to be demonstrated in both to conclude a positive result. Comparisons are based on absolute differences in 6-year incidence from the Kaplan-Meier estimator (with 90% CI), using inverse probability of treatment weighting to account for minimization factors (tumor size and rete testis invasion) as well as the other comparison (modality or frequency of scans).^22^ Standard errors are calculated using bootstrapping.

The primary and key secondary outcomes are also considered in an analysis restricted to relapsing patients. Proportions of relapsing patients with ≥ stage IIC disease (or ≥ 3 cm) are compared between factorial groups using χ^2^ tests. The trial was sufficiently powered to exclude an increase of 38% (to ≥ 76%). Method of relapse detection is also compared using χ^2^ tests; IGCCCG classification is presented but no test was performed because of small numbers. Abdominal mass size at relapse (on the basis of CT) is compared using a Mann-Whitney test.

For DFS and OS, hazard ratios (HRs; with 90% CIs) and 5-year estimates are presented from Cox regression models, adjusting for factors as above. Second primary malignancies are presented by factorial group (no formal test because of low numbers).

In addition to outcome data, surveillance details, timing of relapse, and relapse treatment are described. Median follow-up is reported on the basis of reverse Kaplan-Meier.

## Results

### Patients

A total of 669 participants were enrolled from 35 UK centers (2008-2014), mean age 39 years. Arms were well balanced in terms of key characteristics (Table 1). Mean tumor size was 2.9 cm, 581 (87%) were pT1, and 358 (54%) were low risk.

### Surveillance and Follow-Up

**Fig 2:**
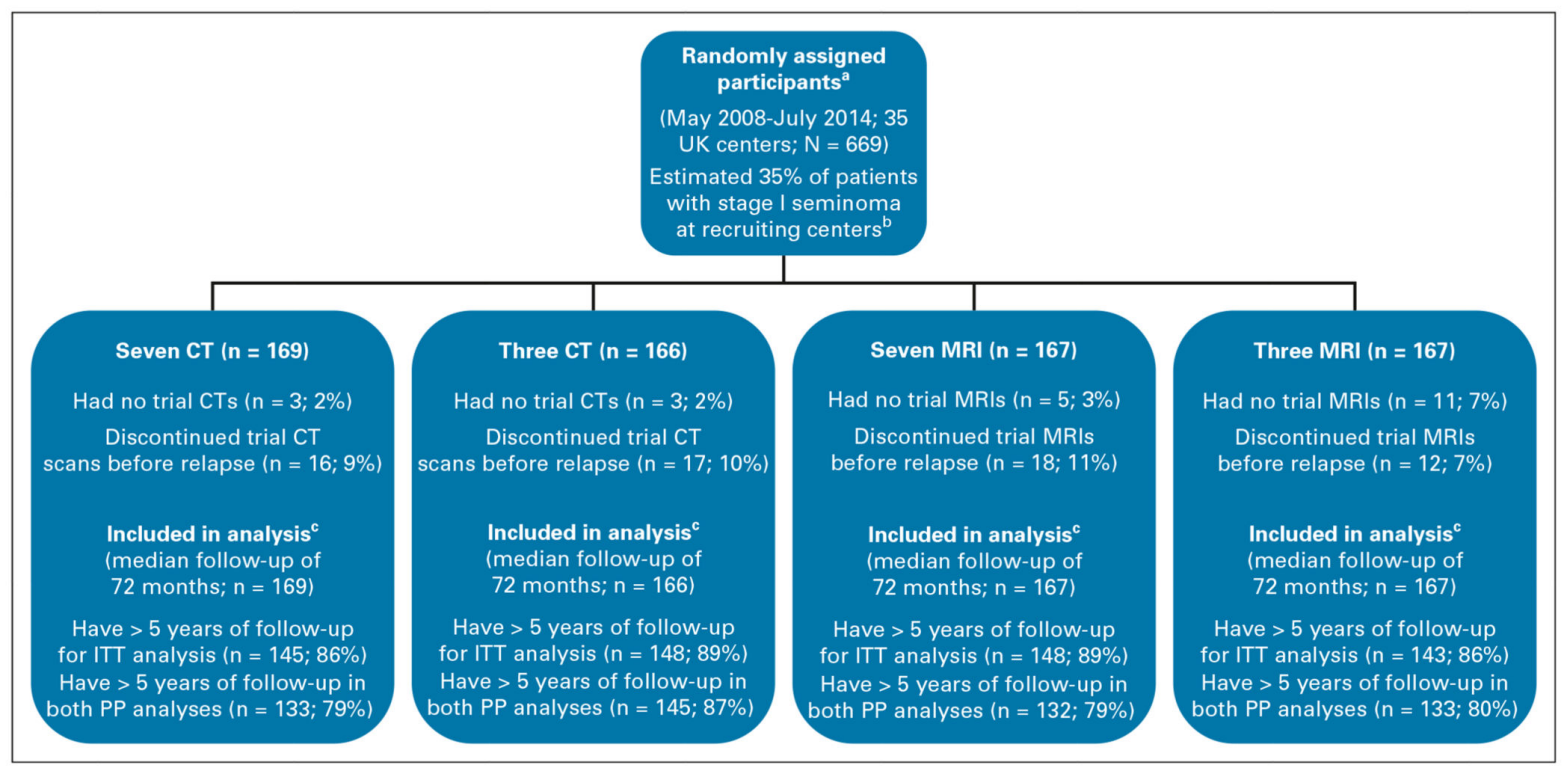
TRISST CONSORT diagram. ^a^One patient prospectively identified as ineligible because of preop and postop AFP being slightly above center ULN (9 and 10 IU/L, respectively; ULN = 7 IU/L), but was allowed to enroll on the basis that these marker values were considered normal for the individual. ^b^On the basis of screening logs completed for a discrete period during trial recruitment. ^c^Analysis of primary and key secondary outcomes is based on time-to-event methods; hence, all patients are included with censoring at the time of being lost to follow-up (or noncompliance in the case of per-protocol analysis, see Appendix Table A1 for per-protocol definitions). AFP, alpha-fetoprotein; CT, computed tomography; ITT, intention-to-treat; MRI, magnetic resonance imaging; PP, per-protocol; ULN, upper limit of normal.

Compliance with allocated schedule was generally good. Eighty-five (13%) participants discontinued early, similar across arms (Fig 2); most commonly, patients were lost to follow-up or moved away (48). Only small numbers were unable to tolerate MRI (seven) or were too large for the scanner (two). Where patients discontinued trial surveillance, data collection continued wherever possible. Median follow-up (including postrelapse) was 72 months with 589 (88%) monitored to 5 years or relapse; the remainder withdrew, were lost to follow-up, or died from another cause before 5 years.

On the basis of PP definitions, the numbers of patients who were compliant throughout the 5-year surveillance period in terms of scan modality were 287 (86%) and 265 (80%) for CT and MRI, respectively; in terms of compliance with scan frequency, the numbers were 278 (83%) and 288 (86%) for seven- and three-scan schedules, respectively.

One hundred nineteen patients had some form of unscheduled imaging likely related to their cancer, which was not prompted by another trial investigation (ie, clinical examination, symptoms, rising markers, or equivocal finding on scheduled imaging; 187 scans). The majority of these were booked in error (118, 63%; most commonly where chest was included on scheduled CT). Others were in patients who had withdrawn from trial surveillance (38, 20%) or were performed to make up for previous missed scans (16, 9%). A further 67 unscheduled scans were reported with no reason given. No trends emerged suggesting systematic differences between arms.

### Primary and Secondary Outcomes: CT Versus MRI

Eighty-two patients (12%) relapsed, 41 in each of the CT and MRI groups. Most were detected on scheduled abdominal imaging (31, 76% CT; 30, 73% MRI) and tended to be identified earlier with MRI (Table 2, Appendix Fig A1, online only). Median abdominal mass size was 2.2 cm in both groups; only one patient (three CT) was IGCCCG intermediate prognosis.^16^ Of those without confirmed relapse, five in each of the MRI and CT groups had one or more equivocal scan.

In total, 10 patients (1.5%) had stage ≥ IIC relapse. There were fewer events in the MRI group (two, 0.6%) compared with the CT group (eight, 2.6%); noninferiority was demonstrated (decrease of 1.9%, 90% CI, –3.5 to –0.3, Table 3A) and confirmed in both PP analysis and analysis on the basis of central review results. Taking the relapsed group as the denominator, this was a decrease of 14.6% (–26.2 to –2.6), from 19.5% with CT to 4.9% with MRI, in the proportion with ≥ IIC disease.

Incidence of tumor size ≥ 3 cm on relapse was 3.6% (24 events). Again, slightly fewer events occurred in the MRI group (11, 3.4%; 13, 4.1% CT; Table 3A). Noninferiority was demonstrated (0.8% decrease, CI –3.3 to 1.7); PP results were similar.

### Primary and Secondary Outcomes: Seven Versus Three Scans

More relapses occurred in the three-scan group (46) compared with seven scans (36), which is unexpected, given that scanning frequency only has the potential to affect timing/stage of relapse. Although the majority were detected by scheduled abdominal imaging in both groups (30, 83% seven scans; 31, 67% three scans), detection by markers or unscheduled imaging was more common with three scans (Table 2). Timing also tended to be slightly later, reflecting schedule, although relapse beyond 3 years was rare (Appendix Fig A1).

There were more ≥ IIC relapses with three scans (9, 2.8%) compared with seven scans (1, 0.3%), a 2.5% increase (1.0%, 4.1%; Table 3B), but within the noninferiority margin (5.7%). Noninferiority was confirmed in PP analysis and analysis on the basis of central review. Taking the relapsed group as the denominator, there was an increase of 16.8% (0.6%, 27.4%), from 2.8% with seven scans to 20.0% with three scans, in the proportion with ≥ IIC disease. This was, again, within the noninferiority margin (38%).

Incidence of tumor size ≥ 3 cm on relapse was 2.0% higher (CI –0.4 to 4.4) with three scans (15, 4.7%) compared with seven (nine, 2.7%), although within the non-inferiority margin (Table 3B). PP results were similar.

Considering outcome events in the four individual trial arms, the difference between three CT and seven CT arms as more marked than for three MRI versus seven MRI (Table 4).

### Relapse Treatment and Long-Term Outcomes

Relapses were treated with low-dose carboplatin and paraaortic radiotherapy (33), combination chemotherapy (normally 4× BEP, 28), or high-dose carboplatin (17; Appendix Fig A2, online only).^23^ Combination chemotherapy was slightly more common in the three-scan group, given the greater number of advanced relapses. Sixty-seven/80 (84%) of patients had a complete response (in one case following surgery); the remainder (13, 16%) had residual mass and normal markers. Two patients were treated for further progression, but none had active disease at the end of follow-up. There were no tumor-related deaths.

Five-year DFS was similar in CT and MRI groups (88% *v* 86%; HR = 1.12; CI, 0.79 to 1.59) and in seven- and three-scan groups (89% *v* 85%; HR = 1.38; CI, 0.97 to 1.97; Appendix Fig A3, online only). Events included seven deaths from other causes. Five-year OS was ≥ 98% for all groups. Nine patients developed secondary malignancies (three prostate, three skin, two lung, and one colon), similar numbers in each arm.

## Discussion

TRISST provides the first multicenter, randomized evidence comparing different imaging modalities and schedules for surveillance of stage I seminoma. Outcomes were excellent in all arms, and survival approached 100% after median 6 years. This confirms observational data showing that surveillance is a safe approach.^24,25^

TRISST was designed to exclude an increase in advanced relapse (RMH stage ≥ IIC) of 5.7% or more associated with the use of MRI or fewer scans. Noninferiority against this criterion was demonstrated in ITT and PP analyses for both comparisons. However, given the lower-than-expected incidence of events, the prespecified noninferiority margin may be less relevant and it is important to consider other aspects of the data to confirm this conclusion.

There were fewer ≥ IIC relapses with MRI compared with CT; 90% CIs exclude the possibility of any increase associated with MRI. Noninferiority was also confirmed with the alternative definition of advanced relapse (≥ 3 cm), where incidence was closer to that expected in the design. Data on relapse timing show a trend toward earlier detection with MRI. Findings align with observational studies indicating that MRI, with experienced radiologists, is a safe alternative in this setting.^26–28^ Numbers of advanced relapses on MRI were too small to assess center variation. Independent central scan review (to be reported separately) will provide further insights into impact of radiologist experience. Although some national guidelines already recommend MRI,^29^ to date, supportive evidence has been insufficient, and is crucial, given the costs associated with MRI. Health economics data (to be reported separately) will facilitate a holistic evaluation of MRI in this setting; it may not be deemed cost-effective unless a reduction in number of scans can also be implemented.

Incidence of ≥ IIC relapse in the seven-scan group was particularly low (one event), making it challenging to perform a statistically valid and relevant noninferiority assessment of three-scan schedules. Although more events occurred in three-scan arms, the absolute number was still low (nine, 2.8%). Furthermore, there were more relapses verall in three-scan arms. Since scan frequency will not affect the number of relapses, this suggests the group was slightly higher risk despite random assignment and apparent balance in terms of known risk factors. Only 4/9 ≥ IIC relapses in the three-scan group could potentially have been identified at an earlier scan with the seven-scan schedule: two at the 12-month scan, one at the 24-month scan, and one at either 48 or 60 months. Given the small number and variation in timing, these do not suggest an obvious modification to improve the three-scan schedule. Treatment/response data are not available for one of these patients; outcomes for the other three were good (one complete response after carboplatin and surgery; one complete response after BEP; one residual mass, normal markers after BEP; none had further progression). It is possible that earlier detection may have avoided use of BEP.

In keeping with other studies,^2,24,25,30^ relapse beyond 3 years was uncommon (5/558 at risk, < 1%). These were not necessarily later-stage relapses (two IIA, two IIB, and one IIC), and only 2/5 were treated with BEP. The TRISST Protocol (online only) included regular marker assessment and clinical examination up to 6 years in all arms, which may be important for detecting the small number of later relapses if scans are stopped earlier.

Considering the alternative advanced relapse definition (≥ 3 cm), the increase associated with the three-scan schedule was small (six events, 2.0% increase, ITT) and noninferiority was demonstrated. Thus, the impact of a less frequent imaging schedule on the selection for the use of local treatment at relapse (either radiotherapy or, as recently reported, minimally invasive retroperitoneal surgery^31,32^) is likely to be small, especially as our data suggest some centers use the same approach (either high-dose carboplatin^23^ or BEP) regardless of relapse stage/size. Hence, numbers of advanced relapses would not affect care. Perhaps, most importantly, outcomes in three-scan arms were excellent, despite more advanced relapses (5-year survival 99%), suggesting no longer-term detriment associated with a reduced schedule. The use of more sensitive biomarkers, such as miRNA-371, in future practice may further reduce the need for frequent scanning.^33^

Although the trial is not powered to assess interactions between scan frequency and modality, it is notable that 8/9 stage ≥ IIC relapses occurred in the three-CT arm, suggesting less impact of reducing the number of scans with MRI. A three-MRI schedule is attractive, avoiding irradiation but limiting increased costs.

As an alternative approach to reduce radiation exposure, a single-arm, prospective study (209 patients with seminoma) has suggested that quality of low-dose CT for this purpose is also acceptable.^34–36^ However, more robust randomized evidence is not yet available.

A limitation of the trial is that the cohort was relatively low risk; only 7% had both Warde risk factors.^20^ Thus, generalizability of findings for this group are less clear. A risk-adapted approach may be appropriate.^37^ However, evidence to validate these risk factors remains limited^38^; further analysis of TRISST data will inform this area.

A limitation of any study assessing technology is their continuing advancements. Both CT and MRI have undergone significant recent developments (specifically, CT dose optimization and MRI image acquisition techniques). A key development over the past 10 years has been diffusion-weighted MRI (DW-MRI). DW-MRI is the most sensitive imaging technique to detect lymph nodes but lacks specificity.^39,40^ However, the superior sensitivity of DW-MRI over other cross-sectional imaging means that detection of retroperitoneal lymph node relapse is best suited to this technique.

Further potential limitations relate to compliance and loss to follow-up. Effectiveness of surveillance relies on good adherence to schedules. Here, 13% did not start their allocated schedule or discontinued early. However, PP analyses indicate that the impact of noncompliance on conclusions was negligible. Follow-up of patients who stopped surveillance continued wherever possible and, with a median follow-up of 72 months, likelihood of missed relapses is small.

In conclusion, surveillance is a safe management approach for stage I seminoma—advanced relapse was rare, salvage treatment successful, and long-term outcomes excellent, regardless of imaging frequency or modality. MRI can be recommended to avoid irradiation. Furthermore, no adverse impact on long-term outcomes was seen with a reduced imaging schedule.

## Supplementary Material

Appendix

## Figures and Tables

**Table 1:** Patient Characteristics at Random Assignment

Patient and Tumor Characteristics	Seven CT	Three CT	Seven MRI	Three MRI	Overall
Age, years					
Mean (SD)	39 (10.1)	38 (9.2)	39 (10.9)	39 (10.0)	39 (10.0)
Range	23-76	19-64	18-64	19-72	18-76
Max. tumor diameter, cm
Mean (SD)	2.9 (1.8)	2.9 (1.7)	2.9 (1.6)	2.9 (1.8)	2.9 (1.7)
≤ 2, No. (%)	71 (42)	62 (37)	53 (32)	69 (41)	255 (38)
2-3, No. (%)	39 (23)	44 (27)	52 (31)	36 (22)	171 (26)
3-4, No. (%)	28 (17)	27 (16)	29 (17)	27 (16)	111 (17)
> 4, No. (%)	31 (18)	33 (20)	33 (20)	35 (21)	132 (20)
Rete testis invasion, No. (%)					
No	110 (65)	111 (67)	109 (65)	109 (65)	439 (66)
Unknown	4 (2)	1 (1)	1 (1)	3 2)	9 (1)
Yes	55 (33)	54 (33)	57 (34)	55 (33)	221 33)
Pagetoid	17 (31)	16 (30)	15 (26)	14 (25)	62 (28)
Interstitial	10 (18)	10 (19)	15 (26)	10 (18)	45 (20)
Not defined	25 (45)	20 (37)	22 (39)	28 (51)	95 (43)
Not known	3 (5)	8 (15)	5 (9)	3 (5)	19 (9)
Warde risk factors, No. (%)
Neither	95 (56)	90 (54)	87 (52)	86 (51)	358 (54)
≤ 4 cm and rete testis invasion	41 (24)	43 (26)	46 (28)	45 (27)	175 (26)
> 4 cm and no rete testis invasion	15 (9)	21 (13)	22 (13)	23 (14)	81 (12)
> 4 cm and rete testis invasion	14 (8)	11 (7)	11 (7)	10 (6)	46 (7)
Unknown (rete testis invasion)	4 (2)	1 (1)	1 (1)	3 (2)	9 (1)
Side of tumor, No. (%)					
Left	76 (45)	83 (50)	83 (50)	82 (49)	324 (48)
R ight	92 (54)	82 (49)	83 (50)	85 (51)	342 (51)
Both	1 (1)	1 (1)	1 (1)	0 (0)	3 (< 1)
T stage, No. (%)
T1	153 (91)	137 (83)	144 (86)	147 (88)	581 (87)
T2	13 (8)	20 (12)	20 (12)	19 (11)	72 (11)
T3	3 (2)	9 (5)	3 (2)	1 (1)	16 (2)
Postoperative LDH,^a^ No. (%)					
In normal range	146 (86)	145 (87)	148 (89)	144 (86)	583 (87)
Raised	15 (9)	16 (10)	10 (6)	17 (10)	58 (9)
Not assessed	8 (5)	5 (3)	9 (5)	6 (4)	28 (4)
Total, No. (%)	169 (100)	166 (100)	167 (100)	167 (100)	669 (100)

Abbreviations: AFP, alpha-fetoprotein; CT, computed tomography; HCG, human chorionic gonadotropin; MRI, magnetic resonance imaging.

a Normal postoperative AFP and Β-HCG were required for eligibility.

**Table 2:** Method of Detection and Tumor Characteristics at Relapse

Method of Detection and Tumor Characteristics	CT	MRI	Seven Scans	Three Scans
First sign of relapse,^a^ No. (%)					
Symptoms	3 (7)	2 (5)	2 (6)	3 (7)
Tumor markers	2 (5)	6 (15)	3 (8)	5 (11)
Scheduled abdominal scan	31 (76)	30 (73)	30 (83)	31 (67)
Unscheduled abdominal CT scan	5 (12)	2 (5)	1 (3)	6 (13)
Scheduled abdominal scan and scheduled chest x-ray^b^	0 (0)	1 (2)	0 (0)	1 (2)
Abdominal mass size on CT, cm^c^
No.	40	37	34	43
Median	2.2	2.2	2.2	2.3
Range	1.0-9.0	1.0-6.2	1.0-5.3	1.0-9.0
IGCCCG intermediate, No. (%)	1 (0)	0 (0)	0 (0)	1 (2)
RMH stage on the basis of CT, No. (%)
IIA	16 (39)	16 (39)	14 (39)	18 (39)
IIB	17 (41)	23 (56)	21 (58)	19 (41)
IIC	3 (7)	2 (5)	1 (3)	4 (9)
IIIA	1 (2)	0 (0)	0 (0)	1 (2)
IIIB	1 (2)	0 (0)	0 (0)	1 (2)
IIIC	1 (2)	0 (0)	0 (0)	1 (2)
IVB	2 (5)	0 (0)	0 (0)	2 (4)
Total, No. (%)	41 (100)	41 (100)	36 (100)	46 (100)

Abbreviations: CT, computed tomography; CXR, chest x-ray; MRI, magnetic resonance imaging; PP, per-protocol; RMH, Royal Marsden Hospital.

a *P* value for CT versus MRI = .342; *P* value for seven versus three scans = .389.

b Both scheduled abdominal MRI and CXR, performed on the same day, were equivocal.

c *P* value CT versus MRI = .853; *P*-value for seven versus three scans = .580.

**Table 3:** Six-Year Incidence of Advanced Relapse According to Factorial Comparison Group

(A) CT *v* MRI			
	Six-Year Incidence, % (No. of events)		
	CT	MRI	Difference,^a^ % (90% CI)
Primary outcome: stage ≥ IIC			
ITT analysis	2.6 (8)	0.6 (2)	-1.9 (-3.5 to -0.3)
PP analysis	2.6 (8)	0.6 (2)	-1.9 (-3.6 to -0.3)
Key secondary outcome: size ≥ 3 cm
ITT analysis	4.1 (13)	3.4 (11)	–0.8 (–3.3 to 1.7)
PP analysis	4.2 (13)	3.4 (11)	–0.7 (–3.3 to 1.8)
Total patients	335	334

(B) Three *v* Seven Scans
	Six-Year Incidence, % (No. of events)		
	Seven Scans	Three Scans	Difference,^b^ % (90% CI)
Primary outcome: stage ≥ IIC			
ITT analysis	0.3 (1)	2.8 (9)	2.5 (1.0 to 4.1)
PP analysis	0.3 (1)	2.9 (9)	2.6 (1.0 to 4.1)
Key secondary outcome: size ≥ 3 cm
ITT analysis	2.7 (9)	4.7 (15)	2.0 (–0.4 to 4.4)
PP analysis	2.7 (9)	4.7 (15)	2.0 (–0.4 to 4.4)
Total patients	336	333	

Abbreviations: CT, computed tomography; ITT, intention-to-treat; MRI, magnetic resonance imaging; PP, per-protocol.

a Incidence with three scans – seven scans (ie, positive values reflect an increase with three scans).

b Incidence with MRI – incidence with CT (ie, negative values reflect a decrease with MRI).

**Table 4:** Six-Year Incidence of Advanced Relapse According to Individual Trial Arm

		Six-Year Incidence, % (No. of events)	
Outcome and Analysis Set	Seven CT	Three CT	Seven MRI	Three MRI
Primary outcome: stage ≥ IIC				
ITT analysis	0 (0)	5.1 (8)	0.6 (1)	0.6 (1)
PP analysis	0 (0)	5.1 (8)	0.6 (1)	0.6 (1)
Key secondary outcome: size ≥ 3 cm
ITT analysis	1.8 (3)	6.4 (10)	3.6 (6)	3.0 (5)
PP analysis	1.8 (3)	6.4 (10)	3.7 (6)	3.1 (5)
Total patients	169	166	167	167

Abbreviations: CT, computed tomography; ITT, intention-to-treat; MRI, magnetic resonance imaging; PP, per-protocol.

## Data Availability

A data sharing statement provided by the authors is available with this article at DOI https://doi.org/10.1200/JCO.21.01199.

## Context

### Key Objective

In the context of computed tomography (CT) surveillance for stage I testicular seminoma, the TRISST trial sought to evaluate whether scan frequency could be reduced, or CT replaced with magnetic resonance imaging (MRI), without an unacceptable increase in advanced relapses requiring intensive treatment.

### Knowledge Generated

This phase III, factorial, noninferiority trial (N = 669) compared (1) seven scans over 60 months versus three scans over 36 months; and (2) CT versus MRI. The number of advanced relapses was very small in all groups (1.5% overall); noninferiority was demonstrated. Relapse treatment was successful, there were no tumor-related deaths, and survival approached 100% in all groups.

### Relevance

In this young patient group who are very unlikely to die from their seminoma cancer, minimizing exposure to potentially harmful radiation is important. TRISST demonstrates that the intensity and duration of CT surveillance can be reduced, or MRI can be used, to reduce irradiation, and that long-term outcomes remain excellent.

## Support

Supported funded by Cancer Research UK (C17084/A8690) and the Medical Research Council Clinical Trials Unit at UCL (MC_UU_12023/28).
