# Supplementary material for: Imaging Modality and Frequency in Surveillance of Stage I Seminoma Testicular Cancer: Results From a Randomized, Phase III, Noninferiority Trial (TRISST)
Source: J Clin Oncol. Author manuscript; Available in PMC 2023 Jun 16. (PMC7614664; doi:10.1200/JCO.21.01199)
Supplement: Appendix [file EMS176874-supplement-Appendix.pdf]

APPENDIX

TABLE A1. Analysis Set Definitions

Analysis Set	MRI *v* CT Comparison	Three- *v* Seven-Scan Comparison
ITT	Includes all patients, analyzed according to their allocated surveillance schedule. For patients who have not experienced an event, observations were censored on the date last confirmed free from advanced disease (normally the most recent trial follow-up assessment)[a]	
PP	MRI: includes all patients who were allocated to one of the MRI arms, with observations censored on the first of the following dates: (1) date of withdrawal from MRI surveillance where this was before receiving any MRI scans (2) date of receiving an abdominal CT surveillance[b] scan (3) date last confirmed free from advanced disease[a] CT: includes all patients who were allocated to one of the CT arms, with observations censored on the first of the following dates: (1) date of withdrawal from CT surveillance where this was before receiving any CT scans (2) date of receiving an abdominal MRI surveillance[b] scan (3) date last confirmed free from advanced disease[a]	Three scan: includes all patients who were allocated to one of the three-scan schedules, with observations censored on the earliest of the following: (1) the first date on which the patient had received either two more or two less than the expected number of abdominal surveillance scans[b], before any relapse[c] (2) date last confirmed free from advanced disease[a] Seven scan: includes all patients who were allocated to one of the seven-scan schedules, with observations censored on the earliest of the following: (1) the first date on which the patient had received either two more or two less than the expected number of abdominal surveillance scans[b], before any relapse[c] (2) date last confirmed free from advanced disease[a]

Abbreviations: CT, computed tomography; ITT, intention-to-treat; MRI, magnetic resonance imaging; PP, per-protocol.

[a]For patients who have been lost to follow-up or withdrawn from trial data collection, follow-up data may include confirmation of disease status obtained through alternative means (eg, general practitioner records).

[b]Surveillance scans are defined as abdominal CTs (or MRIs), including any unscheduled scans that were not clinically indicated (or indication was unclear).

[c]So, for example, at 18 months of follow-up, a patient in one of the 3-scan arms would be expected to have had two abdominal surveillance scans; patients with no scans or with four or more would be censored. A patient in one of the seven-scan arms would be expected to have had three abdominal surveillance scans by this time; patients with one or fewer scans or with five or more would be censored at this time point. A ±2 month window was allowed for the timing of scans relative to the schedule but, where the appropriate number of scans have not taken place within this window, the censor date was the date the scan was due (ie, the 18-month time point in the example given).

Downloaded from ascopubs.org by University College London (ucl) / England on March 29, 2022 from 193.060.240.099
Copyright © 2022 American Society of Clinical Oncology. All rights reserved.

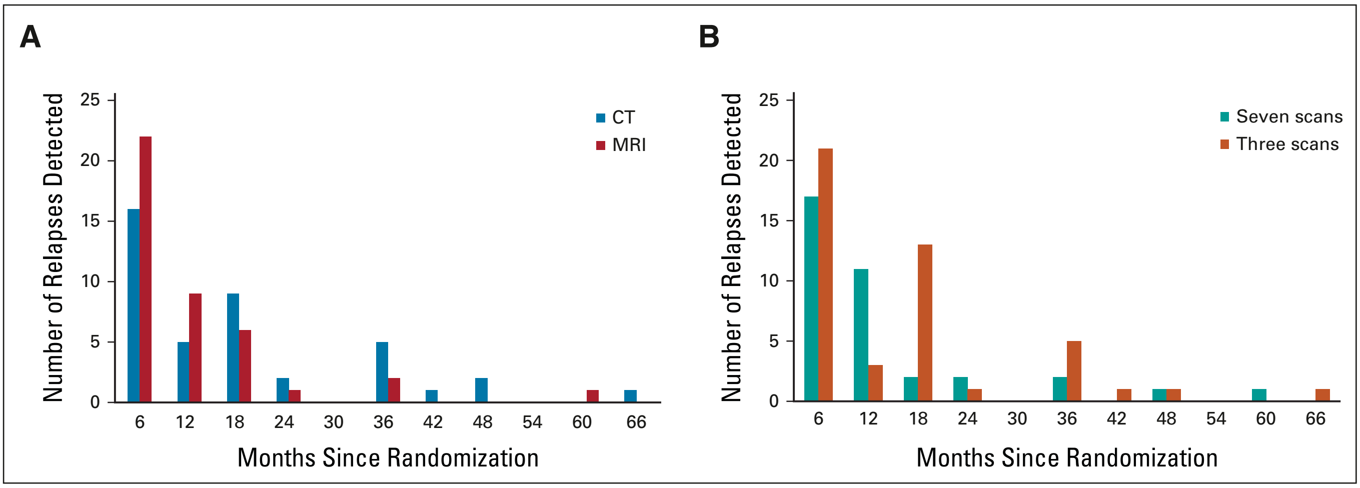

FIG A1. Timing of relapse detection according to allocated scan type (A) and frequency (B). CT, computed tomography; MRI, magnetic resonance imaging.

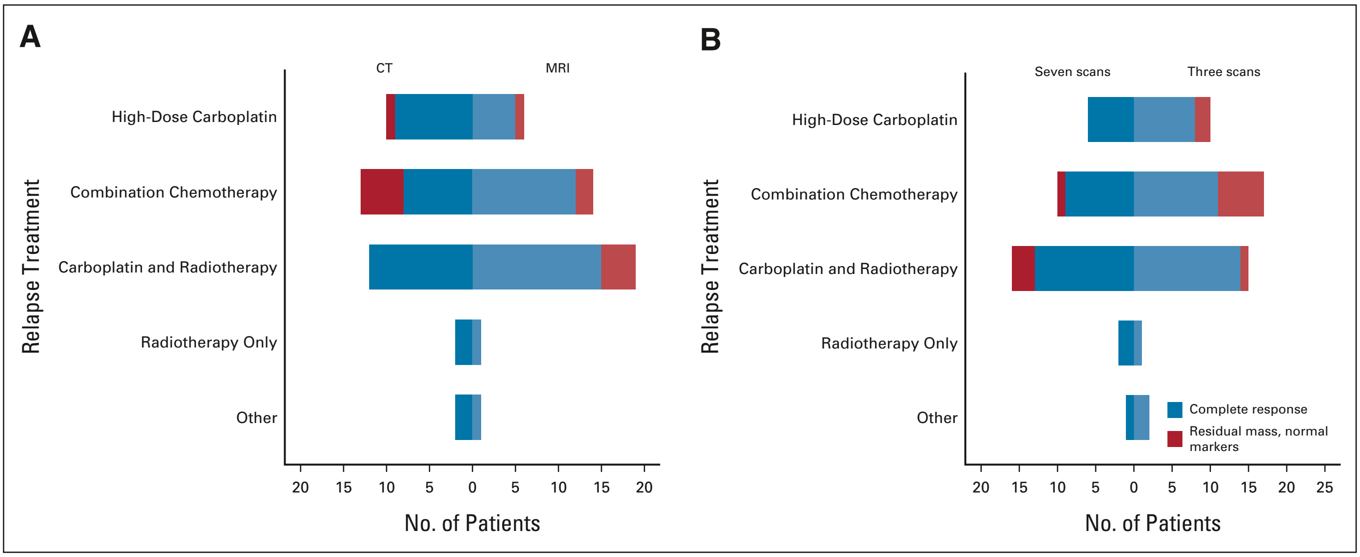

FIG A2. Relapse treatment and response according to allocated scan type (A) and frequency (B). CT, computed tomography; MRI, magnetic resonance imaging.

© 2022 by American Society of Clinical Oncology

Downloaded from ascopubs.org by University College London (ucl) / England on March 29, 2022 from 193.060.240.099
Copyright © 2022 American Society of Clinical Oncology. All rights reserved.

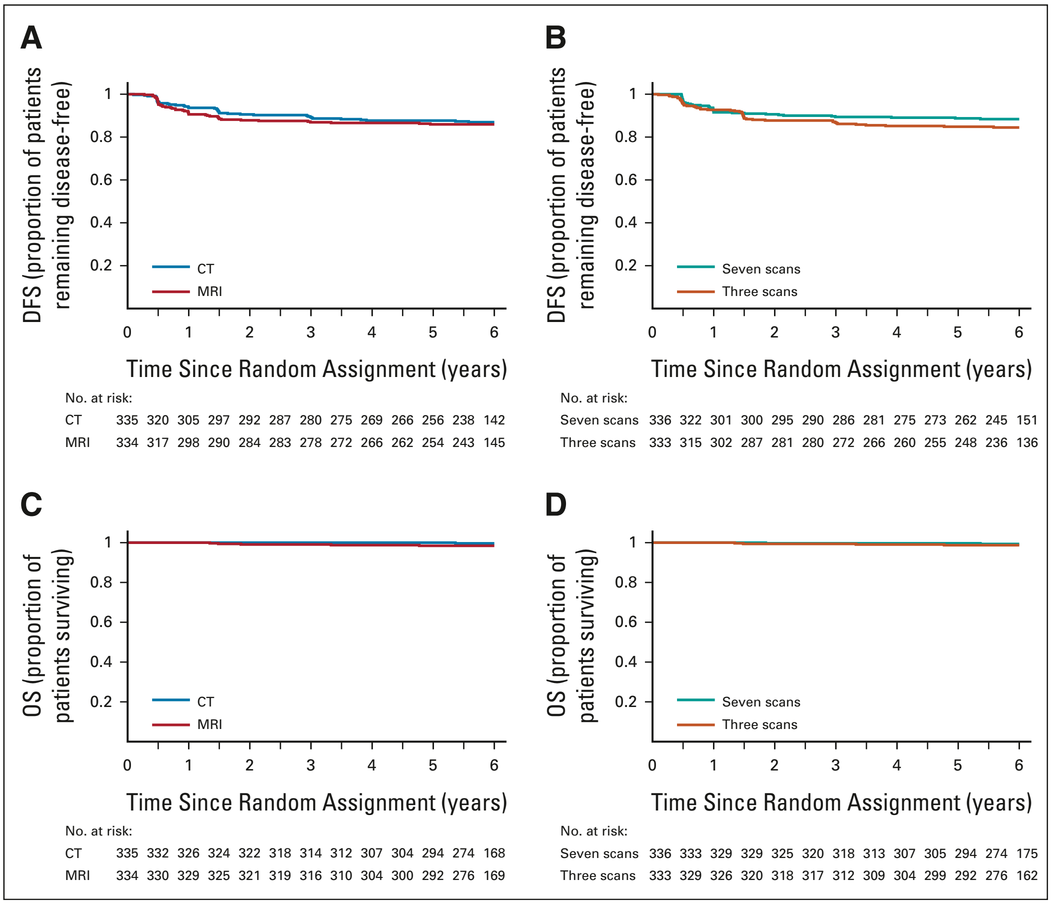

FIG A3. Disease-free and overall survival according to allocated scan type and frequency. CT, computed tomography; DFS, disease-free survival; MRI, magnetic resonance imaging; OS, overall survival.

Downloaded from ascopubs.org by University College London (ucl) / England on March 29, 2022 from 193.060.240.099
Copyright © 2022 American Society of Clinical Oncology. All rights reserved.
